# Co-Amendment of S and Si Alleviates Cu Toxicity in Rice (*Oryza Sativa* L.) Grown on Cu-Contaminated Paddy Soil

**DOI:** 10.3390/ijerph16010057

**Published:** 2018-12-26

**Authors:** Zhihong Lu, Xiao Yan, Zongqiang Wei, Jianfu Wu

**Affiliations:** School of Environmental and Land Resource Management, Jiangxi Agricultural University, Nanchang 330045, China; luzhihong1@163.com (Z.L.); yx198499@126.com (X.Y.)

**Keywords:** Cu contamination, Cu speciation, sulfur, silicon, paddy soil

## Abstract

With irrigation using waste water, application of sewage sludge, and development of mine exploration, copper (Cu) contamination in some paddy fields has become increasingly serious. A greenhouse pot experiment was conducted using a factorial design with three sulfur (S) application rates (i.e., 0, 0.013, and 0.026 g S kg^−1^ soil) and three silicon (Si) application rates (i.e., 0, 0.05, and 0.1 g Si kg^−1^ soil) to test the effect of co-amendment of S and Si on alleviating Cu contamination in paddy soil. There were significant interaction effects between S and Si on soil Cu speciation and Cu uptake by rice plants (except brown rice). Sulfur addition decreased the content of soil-exchangeable Cu, whereas Si addition decreased the content of soil-reducible Cu, suggesting that co-amendment of S and Si generally reduced Cu availability. Copper was biominimized in the soil-rice plant system and rice root had the greatest Cu concentration (163–285 mg kg^−1^). Co-amendment of S and Si decreased the translocation of Cu from soil to rice root, possibly due to decreased soil Cu mobility and enhancement of the formation of iron plaque on rice root. Co-amendment of S-Si at a rate of 0.013 (S)–0.1 (Si) g kg^−1^ soil, respectively, was the optimal among all treatments.

## 1. Introduction

Paddy rice (*Oryza sativa* L.) is one of the most important crops in the world, especially in Asia. In some paddy fields, heavy metals and metalloid contamination (e.g., copper (Cu), arsenic (As), cadmium (Cd)) has become increasingly serious with irrigation using waste water, the application of sewage sludge, and the development of mine exploration. Rice grown on Cu-contaminated paddy soil usually presents inhibition of growth and development, and can accumulate high levels of Cu in rice grain, which may further cause serious problems to human health via food chains [1,2]. Many studies have confirmed that the toxicity of metal(loid)s on plants usually depends on their mobility and availability in soils [3,4]. Generally, the availability of heavy metals and metalloids is associated with soil properties, including pH, organic matter content, iron and manganese oxides, redox status, and clay contents [5]. Among these soil properties, soil pH was found to be the most important factor determining heavy metals and metalloids speciation, solubility, movement, and the eventual bioavailability of heavy metals and metalloids [6].

Sulfur (S) fertilization in paddy soils has received much attention in recent years, as S deficiency dramatically increased due to the increased use of fertilizers with low S content [7,8]. In paddy soils, changes in redox conditions led to S transformation, which can further affect metal(loid)s behavior. Many studies proposed that S can activate metal(loid)s in soil via S oxidation and, therefore, increase their bioavailability [9,10]. Shi et al. suggested that S fertilization was not suitable for Cu-contaminated paddy soil, as it can enhance Cu mobility in rice rhizosphere via oxidation [10]. In contrast, other studies reported that S can promote the formation of iron plaque over root surface, thus sequestering a large amount of metals on root surface and decreasing the bioavailability of metal(loid)s [11,12].

Although silicon (Si) has not been considered as an essential nutrient for plant, Si is beneficial for rice growth, development and resistance against abiotic (metal toxicity, salt and drought stress, nutrient imbalance) and biotic stress (plant diseases and insect pests) [13,14]. Many studies confirmed that the application of Si fertilizer or Si-enriched materials can alleviate the toxicity of heavy metals to rice plants [15,16], and other plants [17]. Overall, S and Si amendments appeared to be potentially useful remediation methods for heavy metal-contaminated paddy soils.

Compared with As and Cd, there is relatively little information with respect to Cu contamination in paddy soil and its corresponding remediation. Moreover, there is a lack of evidence to test the effect of co-amendments of S and Si on alleviation of Cu contamination in paddy soil. The objectives of this study were (1) to investigate Cu speciation and its uptake by rice plant in response to co-amendment of S and Si, and (2) to ascertain an optimal application regime of S and Si for Cu-contaminated paddy soil.

## 2. Materials and Methods

### 2.1. Experimental Design

A greenhouse pot experiment was conducted using a factorial design with three sulfur (finely ground elemental S^0^) application rates (i.e., 0, 0.013, and 0.026 g S kg^−1^ soil) and three silicon fertilizer (Na_2_SiO_3_·9H_2_O) application rates (i.e., 0, 0.05, and 0.1 g Si kg^−1^ soil) at Jiangxi Agricultural University, China (Table 1). In each cylinder pot (radius is 0.15 m and height is 0.25 m), three bunches with three rice seedlings each were transplanted, and N, P_2_O_5_ and K_2_O were applied at the rates of 0.18—0.10—0.18 g kg^−1^ soil. Pots were laid out in a completely randomized design with three replicates, and thus 27 pots were used in this study.

Soil contaminated by Cu was collected from the plow horizon (0–20 cm) of a paddy field near a Cu mine in Fuzhou (28°14’4’’ N, 116°37’’ E), China, for the pot experiment. The soil is classified as Hydragric Anthrosols [18] with silt texture at the plow horizon. The soil had pH (H_2_O) of 4.39, organic matter of 26.91 g kg^−1^, alkali-hydrolyzable nitrogen of 153.12 mg kg^−1^, Olsen phosphorus of 35.61 mg kg^−1^, available potassium (CH_3_COONH_4_-extractable) of 103.50 mg kg^−1^, total Cu of 212.29 mg kg^−1^, available Cu (HCl-extractable) of 88.30 mg kg^−1^, available Si (CH_3_OOH-extractable) of 200.24 mg kg^−1^, and available S (Ca(H_2_PO_4_)_2_-extractable) of 43.43 mg kg^−1^. Fourteen kg air-dried soil (air-dried in greenhouse for about 14 days), which was previously sieved to 3 cm, was used for each pot. Soil total Cu content for each pot was adjusted to 500 mg kg^−1^ by adding CuCl_2_·2H_2_O. Soil having this Cu concentration was considered polluted and not permitted for agricultural land use in China [19]. Then the soil in each pot was submerged and incubated for 7 days before rice seedlings were transplanted. Rice was transplanted on 29 July and harvested on 25 October, 2012. When the rice was harvested, the soil and whole rice plant were collected.

### 2.2. Sample Preparation and Analyses

Rice plants were washed carefully with tap water and deionized water. The grain was air-dried for 17 days to constant weight for grain yield. The grain was further divided into chaff and brown rice for Cu measurement. Subsamples of root, stem, leaf, grain, chaff and brown rice were oven-dried at 80 °C for 4–5 days to constant weight and then ground with a stainless steel grinder to pass through a 100-mesh sieve for the test of Cu concentration. The Cu concentrations of the samples were determined by atomic absorption spectrophotometer (PinAAcle 900T, PerkinElmer, Waltham, MA, USA) after HNO_3_-HClO_4_ digestion. The concentrations of S in different parts of the rice were determined turbidimetrically after HNO_3_-HClO_4_ digestion (759s ultraviolet-visible (UV-Vis) Spectrophotometer, Lengguang, Shanghai, China), and the concentrations of Si in different parts of rice were determined following Elliott and Snyder [20].

Soil of each plot was air-dried for about 14 days and sieved prior analysis. Soil Cu was characterized by a three-stage sequential extraction procedure proposed by the European Communities Bureau of Reference that differentiated four Cu forms and consisted of three extractants: 0.11 M CH_3_COOH, 0.1 M NH_2_OH·HCl (pH = 2), and 1 M CH_3_COONH_4_ (pH = 2) [21]. Briefly, 1.0000 g dry weight soil was combined with 40 mL of each 0.11 M CH_3_COOH (F1 fraction) and 0.1 M NH_2_OH·HCl (pH = 2, F2 fraction) in 50-mL centrifuge tubes. The tubes were successively shaken at 120 rpm for 16 h. The extract was separated from the solid residue by centrifugation at 4000 rpm for 20 min (Centrifuge 5804R, Eppendorf, Hamburg, Germany). After treated with CH_3_COOH and NH_2_OH·HCl, the solid residue was digested by 10 mL 8.8 M H_2_O_2_, and then extracted by 50 mL 1 M CH_3_COONH_4_ (pH = 2, F3 fraction). Subsequently, sample solutions were centrifuged at 4000 rpm for 20 min and supernatants were collected. The remaining solid residues after successive extraction were analyzed for total Cu (i.e., residual Cu, F4 fraction) after digestion by 8 mL concentrated HCl, 5 mL concentrated HNO_3_, 4 mL concentrated HF, and 2 mL concentrated HClO_4_. The Cu concentrations of each extract were determined by an atomic absorption spectrophotometer. The detection limits of Cu was 1.5 μg kg^−1^.

In the sequential extraction procedure, soil phases nominally isolated in F1, F2, F3, and F4 fractions were exchangeable Cu, reducible Cu (e.g., iron/manganese oxides sorbed Cu), oxidizable Cu (organic matter and sulfides associated Cu), and residual Cu, respectively [21].

### 2.3. Statistical Analyses

All analyses were performed in the statistical program R (ver. 3.1.2, R Development Core Team, Vienna, Austria) [22]. Differences in soil Cu content and its uptake by rice plants were analyzed statistically using two-way analysis of variance (*p* < 0.05). The main factors were S and Si treatments and their interactions. Data were checked for normality and homogeneity, and were transformed using log 10 where test assumptions were not met. Multiple comparisons of means were performed by Tukey’s honestly significant difference (HSD) test using the agricolae package for the R software [23]. Correlation analysis was employed to investigate the relationship among soil Cu and Cu uptake by rice plant for all treatments using cor.test function in R base packages.

## 3. Results

### 3.1. Variations of Soil Cu Speciation in Response to S and Si Addition

Generally, F1 Cu accounted for the greatest proportion of total Cu of the paddy soil (31.34–40.18%), followed by F3 Cu (21.10–30.64%), F4 Cu (13.05–27.43%), and F2 Cu (16.48–20.08%). Both F1 and F2 were significantly affected by S, Si amendments, and their interactions (Table 2). Sulfur addition significantly (*p* = 0.0039) decreased Cu concentration of F1 with the lowest value found in S2 treatment (181.88 ± 3.28 mg kg^−1^, Mean ± standard error (SE), *n* = 9), and as a trade-off S addition increased the concentration of F4 fraction (*p* = 0.003). Silicon addition generally increased F2 Cu (*p* < 0.0001) but decreased the concentration of F3 Cu (*p* = 0.0035). The interaction effects between S and Si were found to be statistically significant for all of the four Cu fractions.

### 3.2. The Uptake and Transfer of Cu in Rice Plant as Affected by S and Si Additions

**Figure 1:**
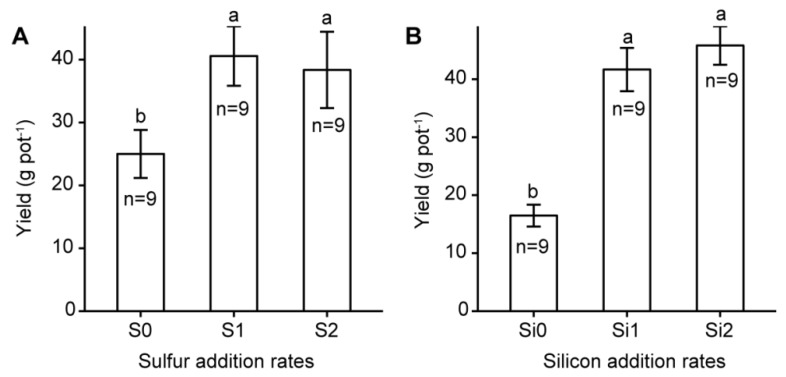
Effect of (**A**) sulfur and (**B**) silicon additions on rice yield. Different letters for each sulfur or silicon addition rate indicate significant differences among different treatments (Tukey HSD test, α = 0.05). Bars represent ±1 standard error (*n* = 9).

**Figure 2:**
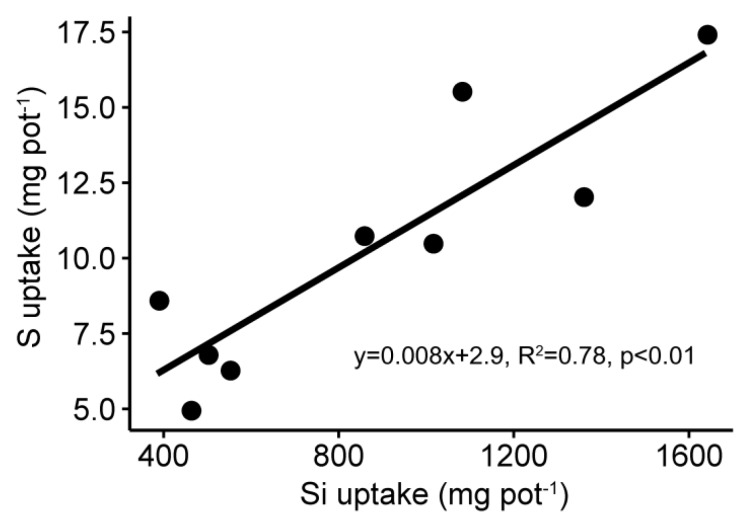
Relationship between silicon uptake and sulfur uptake by above-ground rice. Each point in the graph was the mean value of three replicates.

Both S and Si amendments (in particular Si amendment) significantly (*p* < 0.0001) increased rice biomass and yield, but the effects of interactions between S and Si were not significant (Table 3, Figure 1). In fact, rice yield of Si2 treatment was about three-fold that of treatment without Si addition (46.8 vs. 16.5 g pot^−1^). Co-amendment of S and Si promoted their uptake by above-ground rice and Si uptake was greater than S uptake, as the S addition rate was 26% of Si while its uptake was only about 0.8% of Si uptake (Figure 2).

Copper was not distributed evenly in different parts of the rice plant (Table 3). Rice root had the greatest Cu concentration (163–285 mg kg^−1^), while brown rice had the lowest value which was about 6–11% that of root. In addition, both S and Si amendments significantly decreased Cu concentration in rice root (*p* < 0.0001). Silicon treatment decreased Cu concentration in rice straw (stem and leaf) (*p* < 0.0001), but it had no significant effect on Cu concentration of brown rice.

**Figure 3:**
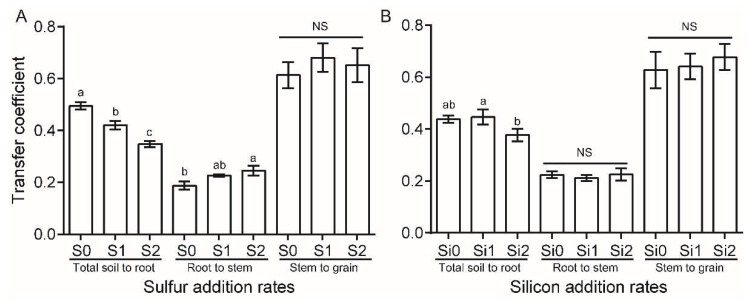
Transfer coefficient of Cu in the soil-root-stem-grain system in response to (**A**) sulfur and (**B**) silicon additions. Different letters on the bars indicate significant differences among sulfur or silicon additions. NS, not significant (Tukey HSD test, α = 0.05). Bars represent ±1 standard error (*n* = 9).

We further investigated the transfer coefficient (TC) of Cu in the soil-root-stem-grain system, calculated as the ratio of the concentrations of one part to the contiguous sub-part in the soil-plant system [24,25]. Generally, TCs of Cu from stem to grain were the greatest (mean 0.65, range 0.46–0.94), followed by those of soil to root (mean 0.42, range 0.30–0.56), and those of root to stem (mean 0.22, range 0.15–0.34) (Figure 3). Both S and Si amendments decreased TCs of Cu from soil to root (*p* < 0.05), but the effect of S appeared to be more significant. Compared with the control, which was free of S, S2 decreased the TC of Cu from soil to root by 30%. Si2 decreased TC of Cu from soil to root by ~15% compared with Si0 and Si1 treatments. TCs of Cu from root to stem tended to increase with S additions (*p* = 0.034). However, S additions had no significant effect on the TC of Cu from stem to grain.

### 3.3. Relationships between Cu in Soil and Its Uptake by Rice Plant

The three extractable Cu fractions (i.e., F1, F2, and F3) were positively inter-correlated with correlation coefficients varied between 0.38 and 0.48 (*p* < 0.05), and these extractable Cu fractions were negatively correlated with residual Cu (*p* < 0.01, Table 4). Among the three extractable Cu fractions, only F1 was positively and significantly correlated with the concentrations of Cu in rice root (*r* = 0.38, *p* < 0.05) and stem (*r* = 0.56, *p* < 0.01). There were no significant correlations between the Cu concentration of brown rice and soil Cu fractions and Cu concentrations of other rice tissues.

## 4. Discussion

**Figure 4:**
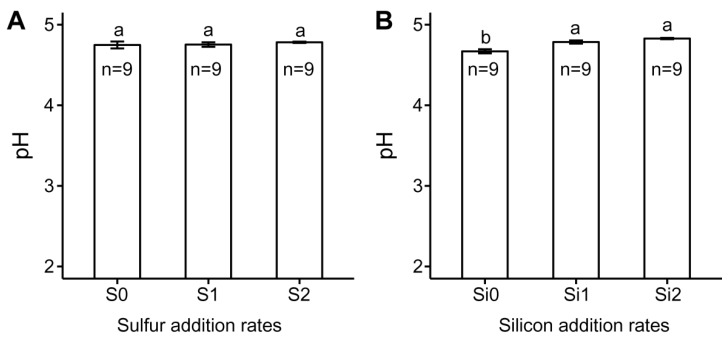
Effect of (**A**) sulfur and (**B**) silicon additions on soil pH. Different letters for each sulfur or silicon addition rate indicate significant differences among different treatments (Tukey HSD test, α = 0.05). Bars represent ±1 standard error (*n* = 9).

Generally, S addition can induce Cu mobilization in soil (i.e., increasing the concentrations of soil exchangeable or extractable Cu), because S oxidation can produce H^+^ ions, thus solubilizing some Cu minerals in soil [9,10]. In our study, F1 Cu (i.e., exchangeable Cu) was not increased by S addition and the S2 treatment had the lowest F1 Cu concentration. In addition, soil pH was not significantly affected by S additions in our study (Figure 4a). These results indicated that S addition did not result in Cu mobilization. This is probably because the S addition rate in our study is relatively lower than other studies, which may limit the S amendment affecting Cu mobilization. For example, Shi et al. conducted a pot experiment with S addition at a rate of 1 g kg^−1^ soil [10], whereas the greatest S addition rate in our study was only 0.026 g kg^−1^ soil. In addition, the soil was always submerged during the rice-growing season, which was not favorable for S oxidation, although the rhizosphere of rice had considerable oxidizing potential [10,26]. This may in turn inhibit the production of H^+^ ions in soil as a result of S oxidation.

As expected, the concentration of Cu in rice root decreased with S addition growing, since soil F1 Cu, the most labile Cu in our study, and TC of Cu from soil to root decreased by S amendments (Table 3, Figure 3). Sulfur addition may decrease the transfer of Cu from soil to rice via other mechanisms. For example, S addition can promote the formation of iron plaque on the surface of rice root, which can hamper Cu uptake by rice [11,12] (see Figure S1, apparent formation of iron plaque can be seen on the surface of the rice root). In our study, we did not study the specific effect of S (and its interactions with Si) on the formation of iron plaque on the rice root. Future studies stressing this issue will be helpful. Collin et al. suggested that the plant can alleviate Cu toxicity via formation of Cu-S compounds (e.g., Cu-cysteine, CuS-inorganic) in stems and leaves [17]. The fact that TCs of Cu from root to stem and Cu concentration of the leaf tended to increase with S additions can be partially explained by the enhanced formation of Cu-S compounds caused by S addition. Investigation of the formation and contents of Cu-S compounds in rice straw in response to S addition is needed in future studies.

The mobilization of soil Cu would decrease with increasing pH due to decreased mineral solubility and increased adsorption [27]. The effects of Si addition on the forms and translocation of soil Cu were partially because Si addition increased soil pH as a result of the hydrolysis of Na_2_SiO_3_·9H_2_O applied in our study (Figure 4b). The fact that soils with Si addition of 0.1 g kg^−1^ soil had the lowest F1 Cu concentration was probably because the soils under this Si addition rate had the highest pH value among the three Si addition rates. The concentration of F2 Cu was significantly increased by Si additions, which indicated Si addition may facilitate the formation of iron/manganese oxides sorbed Cu, as F2 Cu was mainly iron/manganese oxides sorbed Cu [21]. The increase of Cu sorption by iron/manganese oxides was probably due to the increased soil pH as a result of Si application [27].

Sulfur and Si amendments increased rice biomass and yield relative to the treatment without S or Si additions, and the effect of Si appeared to be more pronounced (Figure 1). It is worth noting that, compared with S and Si amendments, the control, which was free of Si, had not received the corresponding dose of sodium because Si was applied as Na_2_SiO_3_·9H_2_O. Sodium is not an essential or beneficial element for rice. It is usually important to halophytic plants or in some low-potassium soils [27]. Soil available potassium, however, was high (103.50 mg kg^−1^) in our study, which was sufficient for rice growing [28]. Therefore, sodium was not a significant contributor to the increases of rice biomass and yield. The fact that S addition increased rice biomass and yield was probably due to S amendment decreasing Cu uptake by the rice plant and thus alleviating Cu toxicity on rice growth. In addition to alleviating Cu toxicity on rice growth, Si is a beneficial element for rice with many beneficial effects on rice growth, thus making it more significant in increasing rice biomass and yield. Consistent with other studies [1,2,29], the highest Cu concentration was observed in rice root and the lowest in brown rice, which suggested Cu had limited transport in the rice plant. In addition, co-amendment of S and Si decreased the TC of Cu from soil to root. Due to the immobilization of Cu in the soil-rice system, Cu concentrations of brown rice for all the treatments were lower than 10 mg kg^−1^, a value above which is not permitted in food sources in China [30]. In this study, co-amendment of S and Si at a rate of 0.013 and 0.1 g kg^−1^ soil, respectively, was optimal among the nine treatments, as the rice yield of this treatment was the highest and complied with the food safety standards of China (Table 3).

## 5. Conclusions

Our data demonstrated that there were significant interaction effects between S and Si on soil Cu speciation and its uptake by rice plants (except the Cu concentration of brown rice). Generally, S and Si amendment decreased the contents of exchangeable Cu or reducible Cu, but increased the contents of residual Cu or oxidizable Cu. Co-amendment of S and Si decreased the translocation of Cu from soil to rice root, possibly because of decreased soil Cu mobility and enhanced iron plaque formation on rice roots. Further investigations of the mechanisms for the interactions between S and Si on alleviating toxicity of Cu-contaminated paddy soil are necessary, such as the formation and contents of iron plaque on rice root and Cu-S compounds in rice straw in response to co-amendment of S and Si.

## Figures and Tables

**Table 1:** Outline description for the fertilization scenarios.

Treatment		Sulfur Addition Rate (g kg^−1^ soil)	Silicon Addition Rate (g kg^−1^ soil)
S0	Si0	0	0
	Si1	0	0.05
	Si2	0	0.1
S1	Si0	0.013	0
	Si1	0.013	0.05
	Si2	0.013	0.1
S2	Si0	0.026	0
	Si1	0.026	0.05
	Si2	0.026	0.1

Sulfur was added as finely ground elemental S^0^, and silicon was added as Na_2_SiO_3_•9H_2_O.

**Table 2:** Effect of sulfur and silicon additions on soil copper speciation. Standard errors of means are given in parentheses.

Treatment		F1	F2	F3	F4
		mg kg^−^^1^			
S0	Si0	187.48 (0.99) ab	87.06 (0.60) d	136.93 (0.51) b	108.30 (1.87) bc
	Si1	200.25 (2.84) a	100.89 (0.83) a	131.71 (2.01) bc	75.84 (1.35) de
	Si2	182.40 (4.94) bc	93.45 (1.05) c	133.97 (8.96) bc	119.39 (11.92) ab
S1	Si0	199.01 (2.30) a	96.99 (0.21) bc	156.62 (2.68) a	69.36 (0.90) e
	Si1	183.90 (1.04) b	88.11 (1.41) d	115.78 (5.09) c	139.55 (3.21) a
	Si2	182.79 (4.91) b	100.50 (0.29) ab	131.72 (0.49) bc	101.94 (3.18) bc
S2	Si0	168.94 (1.34) c	89.12 (0.60) d	133.08 (2.84) bc	139.56 (0.41) a
	Si1	189.04 (0.92) ab	102.79 (0.29) a	142.12 (1.69) ab	93.00 (0.42) cd
	Si2	187.66 (0.35) ab	97.08 (0.55) bc	140.67 (1.53) ab	111.32 (0.72) bc
ANOVA					
S		**	**	NS	**
Si		*	**	**	NS
S×Si		**	**	**	**

Means with different letters within a column are significantly different in response to the interaction effects of sulfur and silicon (Tukey’s honestly significant difference (HSD) test, α = 0.05). *, *p* < 0.05; **, *p* < 0.01; NS, not significant.

**Table 3:** Variations of rice yield and biomass, copper uptake by rice plant, and transfer coefficient of copper in response to the co-amendment of S and Si.

Treatment		Yield	Biomass (DW)	Root	Stem	Grain	Brown Rice
		g pot^−1^	g pot^−1^	mg kg^−1^			
S0	Si0	10.31 (1.42) e	51.93 (4.93) e	241.83 (5.29) bc	59.86 (3.16) a	29.99 (0.78) c	7.59 (0.04) ab
	Si1	29.40 (1.53) cd	60.91 (5.44) e	278.42 (3.16) a	46.87 (0.97) c	24.67 (0.59) e	7.72 (0.56) ab
	Si2	35.32 (0.63) bc	91.19 (4.06) abcd	248.86 (3.06) b	36.51 (0.46) d	29.63 (0.17) c	8.23 (0.63) ab
S1	Si0	22.38 (1.78) cde	72.72 (4.98) cde	242.22 (4.23) bc	58.83 (0.83) a	27.62 (0.22) cd	7.31 (0.02) b
	Si1	46.52 (0.67) ab	96.62 (2.04) abc	233.21 (1.95) c	50.52 (0.38) b	42.20 (0.27) a	7.39 (0.04) b
	Si2	52.85 (2.51) a	117.83 (1.70) a	184.63 (1.39) e	40.47 (1.10) cd	29.76 (0.15) c	7.05 (0.09) b
S2	Si0	16.74 (0.96) de	64.76 (4.79) de	204.96 (3.70) d	36.65 (0.58) d	33.33 (0.30) b	7.90 (0.46) ab
	Si1	49.10 (7.06) ab	89.25 (3.42) bcd	184.61 (1.86) e	46.20 (0.20) c	25.92 (0.80) de	8.06 (0.44) ab
	Si2	49.25 (6.32) ab	106.26 (11.22) ab	163.64 (0.59) f	50.42 (2.31) b	24.34 (0.68) e	9.24 (0.32) a
ANOVA							
S		**	**	**	**		**
Si		**	**	**	**		NS
S×Si		NS	NS	**	**		NS

Means with different letters within a column are significantly different in response to the interaction effects of sulfur and silicon (Tukey HSD test, α = 0.05). *, *p* < 0.05; **, *p* < 0.01; NS, not significant.

**Table 4:** Correlation matrix among various soil Cu forms and Cu concentrations in root, stem, leaf, spike, and grain of rice plant (*n* = 27).

Variable	F1	F2	F3	F4	Root	Stem	Leaf	Chaff	Brown Rice
**F1**	1								
**F2**	0.48 *	1							
**F3**	0.38 *	0.4 *	1						
**F4**	−0.81 **	−0.7 **	−0.67 **	1					
**Root**	0.38 *	−0.23	−0.08	−0.25	1				
**Stem**	0.56 **	−0.13	0.32	−0.41 *	0.17	1			
**Leaf**	0.22	−0.09	0.01	−0.1	−0.25	0.68 **	1		
**Chaff**	−0.17	−0.41 *	−0.31	0.28	0.01	−0.24	0.44 *	1	
**Brown rice**	0.05	0.06	0.11	0.11	−0.31	−0.11	−0.02	−0.32	1

*, *p* < 0.05; **, *p* < 0.01.

## Supplementary Materials

The following are available online at http://www.mdpi.com/1660-4601/16/1/57/s1, Figure S1: Photos of rice seedlings taken at tillering stage grown in a Cu-contaminated paddy soil and treated with increasing doses of sulfur or silicon amendments. S0, S1, and S2 represent sulfur applied at rates of 0, 0.013, and 0.026 g S kg^−1^ soil, respectively, by using finely ground elemental S0; while Si0, Si1, and Si2 represent silicon applied at rates of 0, 0.05, and 0.1 g Si kg^−1^ soil, respectively, by using Na_2_SiO_3_·9H_2_O. Note the reddish rice root due to the formation of iron plaque on it.
