# Supplementary material for: Co-Amendment of S and Si Alleviates Cu Toxicity in Rice (Oryza Sativa L.) Grown on Cu-Contaminated Paddy Soil
Source: Int J Environ Res Public Health. 2018 Dec 26;16(1):57. doi: 10.3390/ijerph16010057 (PMC6339128; doi:10.3390/ijerph16010057)
Supplement: Supplementary file 1 [file ijerph-16-00057-s001.pdf]

Co-amendment of S and Si alleviates Cu toxicity in rice (*Oryza sativa* L.) grown on Cu-contaminated paddy soil

Zhihong Lu, Xiao Yan, Zongqiang Wei*, Jianfu Wu*

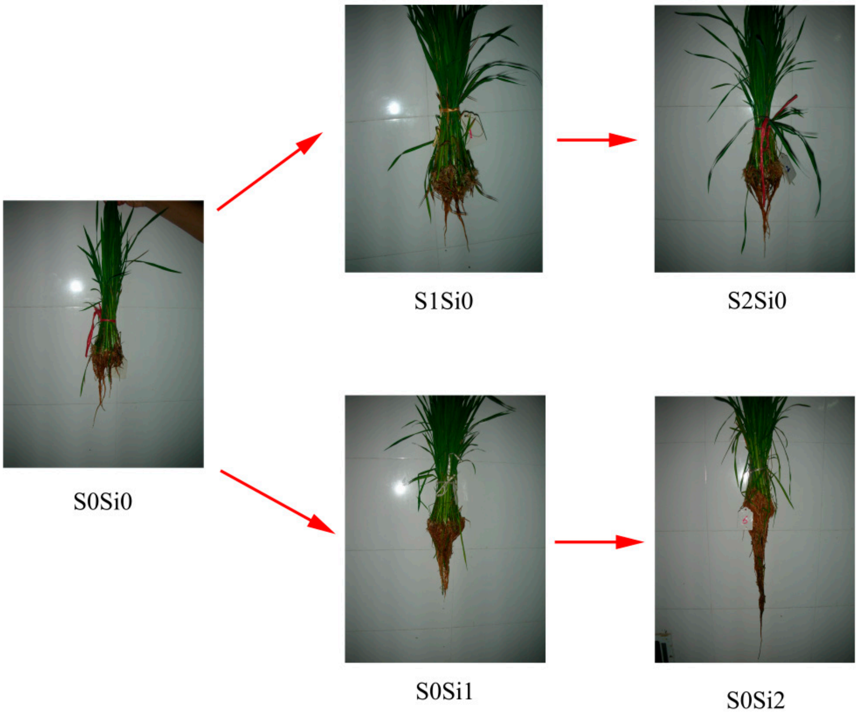

Figure S1. Photos of rice seedlings taken at tillering stage grown in a Cu-contaminated paddy soil and treated with increasing doses of sulfur or silicon amendments. S0, S1, and S2 represent sulfur applied at rates of 0, 0.013, and 0.026 g S kg^{-1} soil, respectively, by using finely ground elemental S0; while Si0, Si1, and Si2 represent silicon applied at rates of 0, 0.05, and 0.1 g Si kg^{-1} soil, respectively, by using $Na_2SiO_3{\cdot}9H_2O$. Note the reddish rice root due to the formation of iron plaque on it.
